# Supplementary figures and images for: DNA metabarcoding uncovers the diet of subterranean rodents in China
Source: PLoS One. 2022 Apr 28;17(4):e0258078. doi: 10.1371/journal.pone.0258078 (PMC9049501; doi:10.1371/journal.pone.0258078)

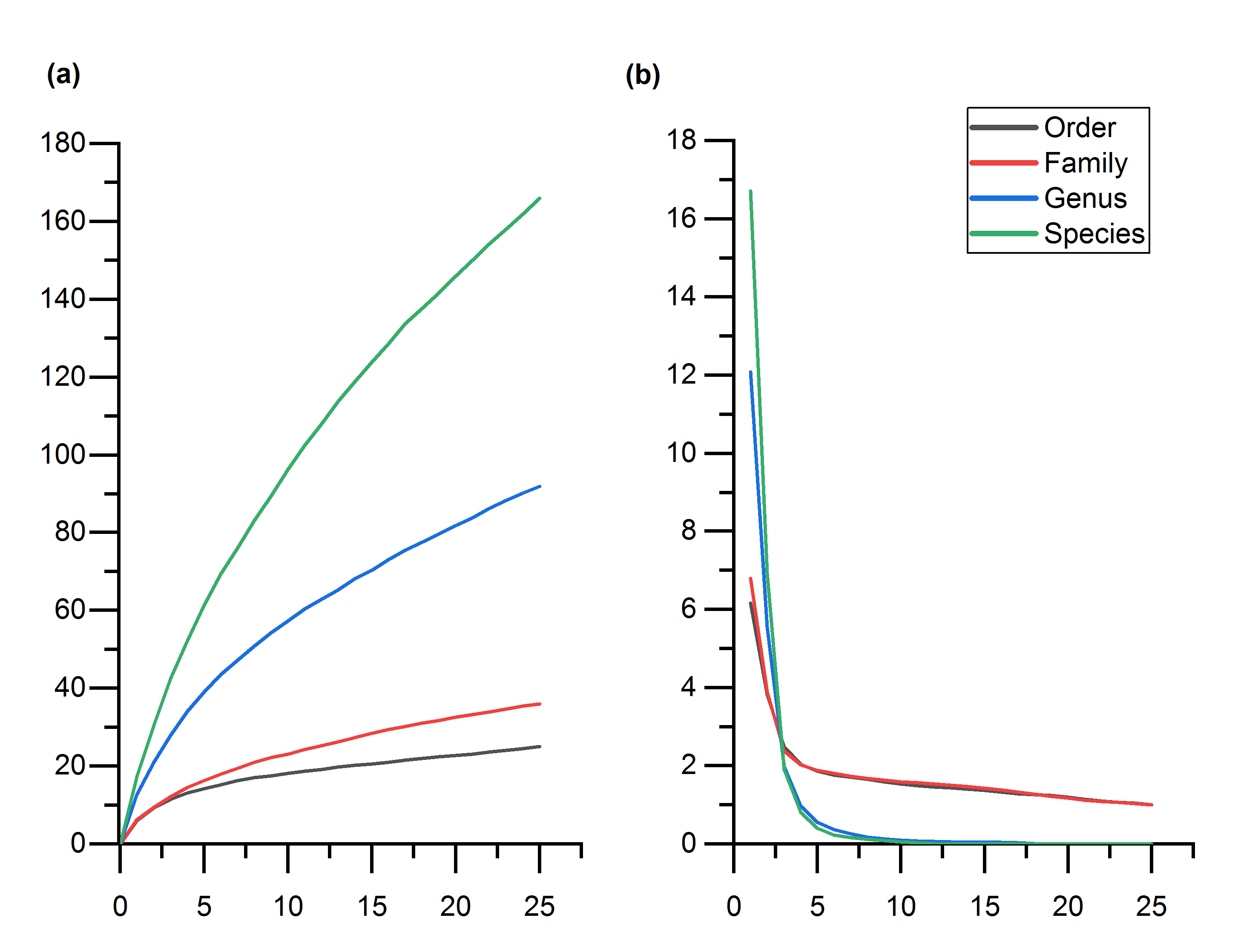

Supplement: S1 Fig — (TIF) [file pone.0258078.s001.tif]

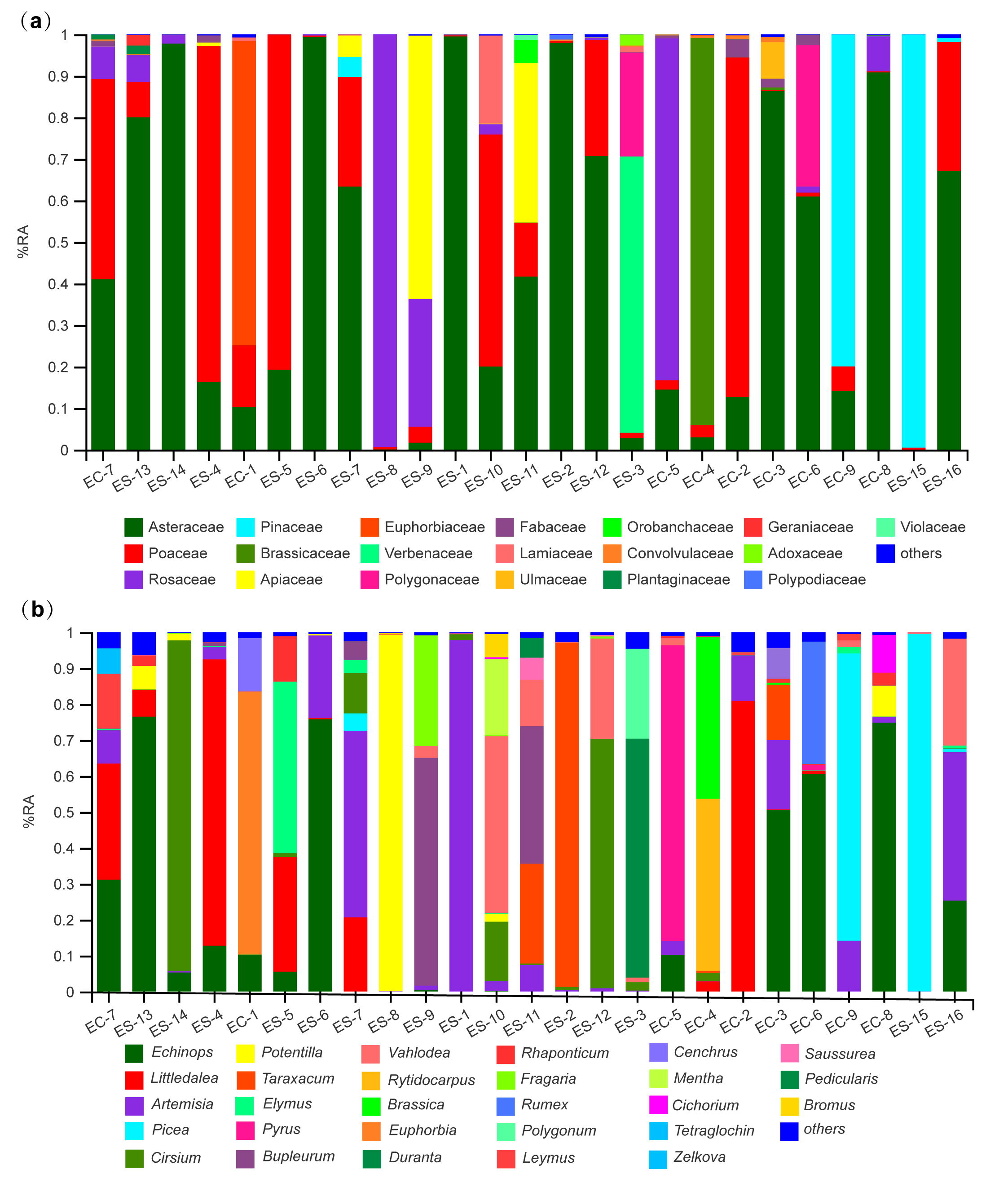

Supplement: S2 Fig — (TIF) [file pone.0258078.s002.tif]

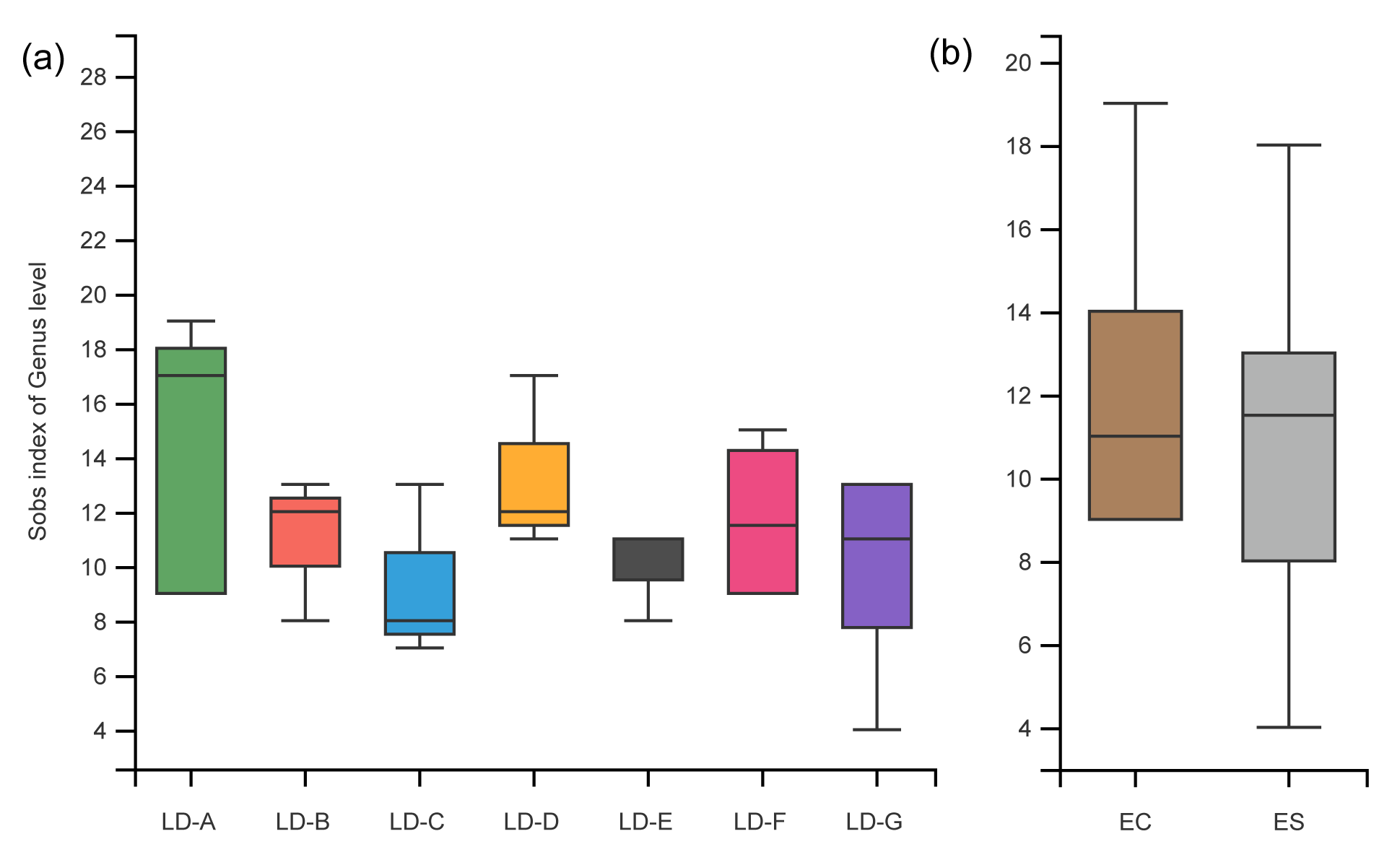

Supplement: S3 Fig — (TIF) [file pone.0258078.s003.tif]
