# Supplementary material for: DNA metabarcoding uncovers the diet of subterranean rodents in China
Source: PLoS One. 2022 Apr 28;17(4):e0258078. doi: 10.1371/journal.pone.0258078 (PMC9049501; doi:10.1371/journal.pone.0258078)
Supplement: Attachment — Submitted filename: Response to Reviewers.docx [file pone.0258078.s008.docx]

January 27, 2022

Dr. Zhang

**Re**: Response for manuscript PONE-D-21-29707 “**DNA metabarcoding Uncover the Diet of Subterranean Rodents in China**”

Dear Dr. Zhang,

Thanks for providing us with this great opportunity to submit a revised version of our manuscript. We appreciate the detailed and constructive comments provided by the reviewers. We have carefully revised the manuscript by incorporating all the suggestions by the review panel.

We hope this revised manuscript has addressed your concerns, and look forward to hearing from you.

Sincerely,

The Authors

**To facilitate this discussion, we first retype your comments in italic font and then present our responses to the comments.**

**Encl. Responses to the comments from Editor.**

**Reply to Editor**

**Additional requirement #1**

*Please ensure that your manuscript meets PLOS ONE's style requirements, including those for file naming. The PLOS ONE style templates can be found at https://journals.plos.org/plosone/s/file?id=wjVg/PLOSOne_formatting_sample_main_body.pdf and*

[*https://journals.plos.org/plosone/s/file?id=ba62/PLOSOne_formatting_sample_title_authors_affiliations.pdf*](https://journals.plos.org/plosone/s/file?id=ba62/PLOSOne_formatting_sample_title_authors_affiliations.pdf)*”*

**Response to additional requirement #1.**

Our manuscript was written with the LaTeX provided by PLOS template, and we made changes to the template before submitting the manuscript. However, there is still a gap between the style of the officially published article, so we have carefully checked our manuscript to ensure that the revised version conforms to the format requirements of your journal to the maximum extent.

**Additional requirement #2**

*We note that the grant information you provided in the ‘Funding Information’ and ‘Financial Disclosure’ sections do not match.* *When you resubmit, please ensure that you provide the correct grant numbers for the awards you received for your study in the ‘Funding Information’ section.*

**Response to additional requirement #2.**

This study was supported by Ecological Adaptation and Molecular Basis of spreading epidemic of Major biological hazards in Forest(2017YFD0600103–4-3), a National Key R & D project of Ministry of Science and Technology, PRC. The funders had no role in study design, data collection and analysis, decision to publish, or preparation of the manuscript.

**Additional requirement #3**

*We note that you have stated that you will provide repository information for your data at acceptance. Should your manuscript be accepted for publication, we will hold it until you provide the relevant accession numbers or DOIs necessary to access your data. If you wish to make changes to your Data Availability statement, please describe these changes in your* ***cover letter*** *and we will update your Data Availability statement to reflect the information you provide.*

**Response to additional requirement #3.**

All of our sequencing data have been uploaded to the NCBI database when we first submitted the manuscript, Accession Numbers are PRJNA753876. You can view my submitted Accession Numbers to access our data. Our previous statement does not affect the availability of our sequencing data.

**Additional requirement #4**

*PLOS requires an ORCID iD for the corresponding author in Editorial Manager on papers submitted after December 6th, 2016. Please ensure that you have an ORCID iD and that it is validated in Editorial Manager. To do this, go to ‘Update my Information’ (in the upper left-hand corner of the main menu), and click on the Fetch/Validate link next to the ORCID field. This will take you to the ORCID site and allow you to create a new iD or authenticate a pre-existing iD in Editorial Manager. Please see the following video for instructions on linking an ORCID iD to your Editorial Manager account:* [*https://www.youtube.com/watch?v=_xcclfuvtxQ*](https://www.youtube.com/watch?v=_xcclfuvtxQ)

**Response to additional requirement #4.**

According to your suggestion, we have updated the ORCID account of the corresponding author in the submission system. The ORCID account is **0000-0002-0127-1254**.

**Additional requirement #5**

*Please amend the manuscript submission data (via Edit Submission) to include author’s ,Yao Zou,Xiaoning Nan.*

**Response to additional requirement #5.**

I am very sorry that I did not find the place to add author information you pointed to in the submission system. I'm here to give a response to that question. In the revision stage, the author Xuxin Zhang sought the help of the authors who were not in the manuscript, so the author expected to reflect their contributions in the revised version.

The **latest author** information is as follows: Xuxin Zhang, Master (529907210@qq.com); Yao Zou, Ph.D.(1179520342@qq.com); Xuan Zou, Master, (3199488932@qq.com); Zhenggang Xu, Ph.D.( xuzhenggang@nwafu.edu.cn); Xiaoning Nan*, Ph.D.( 358727493@qq.com); Chongxuan Han*, Ph.D.( 1139186993@qq.com). All the authors are affiliated to the Key Laboratory of National Forestry and Grassland Administration on Management of Western Forest Bio-Disaster, College of Forestry, Northwest Agriculture and Forestry University, Yangling712100, China.

XXZ,YZ,XNN and CXH contributed to the study design. Sample collection and data analysis were completed by XXZ,YZ,XNN. Experiment was performed by XXZ, YZ and XZ. The first draft was written by XXZ. Revised suggestions were made with the help of ZGX, XNN and CXH. All authors have read and approved the final manuscript.

**Additional requirement #6**

*We note that you have included the phrase “data not shown” in your manuscript. Unfortunately, this does not meet our data sharing requirements. PLOS does not permit references to inaccessible data. We require that authors provide all relevant data within the paper, Supporting Information files, or in an acceptable, public repository. Please add a citation to support this phrase or upload the data that corresponds with these findings to a stable repository (such as Figshare or Dryad) and provide and URLs, DOIs, or accession numbers that may be used to access these data. Or, if the data are not a core part of the research being presented in your study, we ask that you remove the phrase that refers to these data.*

**Response to additional requirement #6.**

We looked closely at the previous version of the manuscript and there were indeed three phrases "**data not shown**". The data in the first and third parts of the paper are not shown as the core data of the conclusion, so we are going to remove them in the revised draft. The data in the second part of the paper will be supplemented in the revised version.

**Encl. Responses to the comments from Reviewer 1.**

**Reply to Reviewer 1**

Dear Reviewers,

Thank you very much for your time involved in reviewing the manuscript and your very encouraging comments on the merits.

We also appreciate your clear and detailed feedback and hope that the explanation has fully addressed all of your concerns. In the remainder of this letter, we discuss each of your comments individually along with our corresponding responses.

To facilitate this discussion, we first retype your comments in italic font and then present our responses to the comments.

**Major comment #1:**

*The hypotheses of the present study were not proposed properly in the introduction. you give detailed introduction on methods, however, you are not aim to update the method used in this study.*

**Response to Reviewer Major comments #1.**

As you mentioned, my introduction gives too much introduction to the method, but it is not very relevant to my research purpose. We revised the introduction according to your suggestion. The introduction of the experimental method is appropriately brief, enrich the description of the research hypothesis and purpose.

**Major comment #2:**

*A map to show the localities of samples is lacking in the present version.*

**Response to Reviewer Major comments #2.**

According to your suggestion, we have added the map showing the sample location, which is **Figure 1** in the revised draft.

**Major comment #3:**

*It is unclear how did you identify these animals, and whether their live in different micro-habitat. It is better to provide pictures to show their differences in morphology and in habitat selection.*

**Response to Reviewer Major comments #3.**

Zokor is a kind of subterranean rodents endemic to East Asia. In this study, two species of zokor were located in Longde County, Ningxia Hui Autonomous Region, China. In this area, we found that the two zokor species (*Eospalax smithii* and *Eospalax cansus*) were sympately distributed, and there were no significant differences in their micro-habitat. The table below lists the three morphologic differences between the two species of zokor, namely, the colour of their coat, the presence or absence of tail hair and the shape of their snout.

|  |  |
| --- | --- |
| *Eospalax smithii* | *Eospalax cansus* |
| The coat is grayish brown | The coat is tawny |
| The tail is short with bushy short hair | The tail is long with sparse white short hairs |
| The snout has an upward cusp | The snout has a smooth upward projection |

**Major comment #4:**

*Authors mentioned ‘ The diversity of zokor’s diet differs greatly among different study areas’, but no figure was given to show these differences.*

**Response to Reviewer Major comments #4.**

In line 319 of the previous version, we mentioned the conclusion 'The diversity of Zokor's Diet Greatly among Different study Areas', but did not give the corresponding picture or table to support this conclusion. After careful examination, we found, regrettably, that this conclusion was not expressed correctly; according to **Table 3**, there was no significant difference in food diversity between the zokor species. We apologize for our oversight. We have deleted this conclusion in the revised version.

The real takeaway is that the zokor's diet is similar to that of the plants in their study area.

**Major comment #5:**

*Authors mentioned ‘there was no significant difference between Family and Genus and among different species of zokor’ without give a picture or table. However, this should be one of the major conclusions of this study. I suggest to give pictures or well labeled tables to support your results in this section*

**Response to Reviewer Major comments #5.**

In the previous version, we mentioned "There was no significant difference between Family and Genus and among different species of Zokor". You suggest giving pictures or tables.

We carefully checked this part, and the corresponding content in the revised version is "**Fig 6**". In this reanalysis, we found that, not that there were no significant differences as previously suggested, but that there were certain types of food that differed significantly between the study area and the zokor species.

In this analysis, the top 10 foods in relative abundance (%RA) were examined for differences between groups. Within groups of different study areas, three foods showed significant differences between study areas (***Littledalea*, *Picea* and *Vahlodea***) (**Fig 6a**), while most other food types showed no differences. Picea, for example, was only present in the diet of LD-B and LD-G study areas, and LD-G was significantly higher than LD-B, which was consistent with the abundance of plant resources in the study area. In LD-G, there was a spruce nursery, while in LD-B, there was a small amount of spruce. From the side, the food resources in different study areas are consistent with those in the study area.

In the comparison between the two zokor species, only ***Cirsium*** showed significant difference among the top 10 food species in %RA (**Fig 6b**), and the intake of ***Cirsium*** by *Eospalax smithii* was significantly higher than that by *Eospalax cansus*. Further analysis showed that 1 *Eospalax cansus* (1/9)and 8(1/2) *Eospalax smithii* had eaten ***Cirsium***. Whether *Eospalax smithii* prefers thistle more than *Eospalax cansus* or whether it is related to the abundance of this plant in its habitat needs further study.

**Major comment #6:**

*PCA results are quite important in this paper, it need to be placed in the major section of this ms.*

**Response to Reviewer Major comments #6.**

In the previous version, we only performed PCA (principal component analysis) in the beta diversity analysis. Since most of the sample points in the PCA graph were clustered together and did not show good grouping differentiation, the images for PCA were included in the supporting figures (Fig.S2).

In the revised version, after careful re-analysis, PCoA (principal coordinate analysis) was added to illustrate the food similarity of zokor in different groups. We named the remade image "Fig.5" and it will appear in the body of the manuscript.

In **Fig 5**, we chose a new level of analysis (genus), and we zoomed out the images representing each sample point, which did not overlap as much as the previous version of the figure. Comparing Fig. 5a & 5b (PCA) and Fig. 5c & 5d (PCoA), we found that there were differences between regions in food similarity, but no differences between the two zokor species. The comparison between the two groups may suggest that the difference in diet may be related to the food resources in the habitat whereas the zokor species' choice does not seem to differ.

**Major comment #7:**

*Figures were not compiled properly, and present as separate small figures in this version. I suggest authors to compile small figures to one big plate by using adobe illustrator.*

**Response to Reviewer Major comments #7.**

Thank you for your constructive comments. In the revised version, we reviewed all the images, added the study area map and zokor identification figures you mentioned in **Comments 2 and 3**, and compiled the same type of figures into one big plate using Adobe illustrator for the rest of the figures. At the editor's suggestion, we also uploaded the figures to the **PACE** website to meet the PLOS ONE publication requirements.

**Major comment #8:**

*Legends for figures and tables are too simple to understand them correctly, abbreviations for figures and tables were not given in the current version.*

**Response to Reviewer Major comments #8.**

According to your suggestion, we will supplement the abbreviations of pictures and forms in the revised version.

**Major comment #9:**

*I suggest you consult a person who is native in English to go through the ms.*

**Response to Reviewer Major comments #9.**

After the initial submission, the editor has suggested that we consult the scientific editor to revise the manuscript's language and spelling. We chose Letpub(https://www.letpub.com/) to edit and revise our manuscript. LetPub is the flagship editorial services brand of Boston-based Accdon LLC. LetPub specializes in editorial services for the scholarly publishing community and provides research communications services, such as a scientific illustration, graphical and video abstracts, and plain language summaries. The manuscripts are edited by native English speakers.

But, to be honest, the language of my manuscript still needs refining. In the revised version, we consulted LetPub and asked them to further edit my manuscript. At the same time, we also consulted Dr. Zhenggang Xu from our team, who has writing experience of more than 30 English papers and provided great help to my revised version.

We would like to take this opportunity to thank you for all your time involved and this great opportunity for us to improve the manuscript. We hope you will find this revised version satisfactory.

Sincerely,

The Authors
